# Co-infection with human polyomavirus BK enhances gene expression and replication of human adenovirus

**DOI:** 10.1007/s00705-018-3810-1

**Published:** 2018-03-26

**Authors:** Iwona Bil-Lula, Mieczysław Woźniak

**Affiliations:** 1 Department of Clinical Chemistry, Wroclaw Medical University, Borowska 211A Street, 50-556 Wrocław, Poland; 2 Department of Pharmacology, College of Medicine, University of Saskatchewan, Saskatoon, Canada

## Abstract

Immunocompromised patients are susceptible to multiple viral infections. Relevant interactions between co-infecting viruses might result from viral regulatory genes which trans-activate or repress the expression of host cell genes as well as the genes of any co-infecting virus. The aim of the current study was to show that the replication of human adenovirus 5 is enhanced by co-infection with BK polyomavirus and is associated with increased expression of proteins including early region 4 open reading frame 1 and both the large tumor antigen and small tumor antigen. Clinical samples of whole blood and urine from 156 hematopoietic stem cell transplant recipients were tested. We also inoculated adenocarcinomic human alveolar basal epithelial cells with both human adenovirus 5 and BK polyomavirus to evaluate if co-infection of viruses affected their replication. Data showed that adenovirus load was significantly higher in the plasma (mean 7.5 x 10^3^ ± 8.5 x 10^2^ copies/ml) and urine (mean 1.9 x 10^3^ ± 8.0 x 10^2^ copies/ml) of samples from patients with co-infections, in comparison to samples from patients with isolated adenovirus infection. *In vitro* co-infection led to an increased (8.6 times) expression of the adenovirus *early region 4 open reading frame* gene 48 hours post-inoculation. The expression of the *early region 4 open reading frame* gene positively correlated with the expression of BK polyomavirus *large tumor antigen* (r = 0.90, p < 0.0001) and *small tumor antigen* (r = 0.83, p < 0.001) genes. The enhanced expression of the *early region 4 open reading frame* gene due to co-infection with BK polyomavirus was associated with enhanced adenovirus, but not BK polyomavirus, replication. The current study provides evidence that co-infection of adenovirus and BK polyomavirus contributes to enhanced adenovirus replication. Data obtained from this study may have significant importance in the clinical setting.

## Introduction

Recipients of hematopoietic stem cell transplantations (HSCTs) are prone to multiple opportunistic infections [1]. Human adenoviruses (HAdV) (taxonomic classification: genus *Mastadenovirus* family *Adenoviridae*) play a significant role in the reactivation of adenoviral infections during the early, post-transplant period [2–4]. They also can be transmitted from a positive donor [5, 6]. Although HAdV infections in immunocompetent subjects are usually asymptomatic and self-limiting, the recipients of HSCTs and other immunocompromised patients present a broad spectrum of clinical manifestations. They can cause a range of infections from asymptomatic to localized or disseminated [3], including acute respiratory disease [7, 8], keratoconjunctivitis [8], pharyngeal–conjunctival fever [1] and hemorrhagic cystitis [7, 9]. Less frequent complications are arthritis, encephalitis [10, 11], gastroenteritis [12, 13] and hepatitis [7, 14].

In transplants a lack of immunological competence caused by chemotherapy and conditioning procedures results in lymphopenia which, in turn, increases patient`s risk of *de novo* infection or the reactivation of a latent virus as well as co-infections with two or more pathogens. Simultaneous infections of adenoviruses and BK polyomavirus (BKPyV), human cytomegalovirus (HCMV), Epstein–Barr virus (EBV) or respiratory syncytial virus (RSV) have been widely described in patients with hematological malignances [8, 13, 15–20]. In our previous work we also showed that co-infection of HAdV and BKPyV is a common complication in patients undergoing HSCTs [21]. Examination of multiple risk factors confirmed that BKPyV infection, transplantation from the matched unrelated donor, and low CD3 + antigenic status (marker of lymphocytes T) increase the risk of HAdV infection [21].

Relevant interactions between co-infecting viruses might result from regulatory genes which trans-activate or repress the expression of other genes. Trans-activating viral proteins may affect the gene expression of host cells [22] as well as the genes of co-infecting virus [23]. In 1992 Moch et al. showed a strong trans-activation of the human cytomegalovirus major immediate-early enhancer by human T-cell leukemia virus type 1 (HTLV-1) [24]. Two years later, Metvalf et al. (1994) reported that the adenovirus E1A 13S gene product (an early protein 1A) up-regulates the cytomegalovirus major immediate early promoter [25]. Going further, in 1997 Kristoffersen et al. reported that both HCMV and BKPyV encode gene products with the inherent potential of acting as heterologous trans-acting factors for the expression of cellular or viral genes. They also demonstrated that BKPyV is able to enhance the expression of HCMV immediate early (IE) promoter in semi-permissive cells [26]. Taking into account our previous observations showing that BKPyV infection increased the risk of HAdV infection in multiple risk factors analysis [21], we hypothesized that infection of BK polyomavirus may facilitate the replication of HAdV in host cells. For this reason the specific aim of the current study was to verify if the expression of the adenovirus E1 promoter is influenced by BKPyV replication and if there is a correlation between polyoma *TAg/tAg* (large tumor antigen/small tumor antigen) and adenoviral *E4orf1* (early region 4 open reading frame 1) expression. Here we demonstrate that replication of HAdV5 in A549-permissive cells (adenocarcinomic human alveolar basal epithelial cells) is enhanced following co-infection with BKPyV and correlates with enhanced expression of genes for both the large and small t antigens.

## Materials and methods

### Patients and clinical samples

We retrospectively examined 156 patients (1-45 years range) undergoing hematopoietic stem cell transplantation at the Department and Clinic of Haematology, Blood Neoplasms, and Bone Marrow Transplantation and the Department of Paediatric Bone Marrow Transplantation, Oncology and Hematology in Wrocław. Written informed consent was obtained for collection of blood from all study participants and the protocol was approved by local Ethics Boards. All recipients were tested for HAdV and BKPyV infection on a regular basis after transplantation. Clinical samples of whole blood and urine were collected. Virological surveillance was performed repeatedly, according to valid protocols for post-transplantation care. The samples of whole blood (EDTA) and urine were centrifuged at 900 x g for 20 min and 900 x g for 10 min, respectively, before freezing at − 20 °C. DNA was extracted from plasma samples using spin columns from the QIAamp Blood Mini Kit (Qiagen GmbH, Hilden, Germany), and from urine using a Syngen Viral Mini Kit PLUS (Syngen, Poland), according to the manufacturer’s instructions.

### A549 culture collection

A549 cells obtained from American Type Culture Collection (ATCC, Manassas, Virginia) (CRM-CCL-185) were propagated in 25 cm^2^ cell culture flasks (Greiner, Germany) in DMEM (Dulbecco’s Modified Eagle’s Medium, Sigma Aldrich, USA) supplemented with 5% of FBS (fetal bovine serum, Sigma Aldrich) and 1% of penicillin-streptomycin (Sigma Aldrich) under stringent aseptic conditions, at 37 °C, 5% CO_2_ for 3–4 days. 3 passages were performed before seeding of cells for particular experiments to increase the strength of the cells. The cells harvested from the flask with 0.25% Trypsin-EDTA solution (Sigma Aldrich) were used for further experiments.

### HAdV5 and BKPyV propagation

Prototype strains of BKPyV (VR-837) were obtained from the ATCC while HAdV5 was provided by the Institute of Immunology and Experimental Therapy, in Wroclaw. The viruses were grown on A549 cells in 5% DMEM supplemented with 5% FBS and 1% of antibiotics, under stringent aseptic conditions, at 37 °C, 5% CO_2_ for 3-14 days. The viruses were cultured repeatedly to obtain homogenous virus stocks. After centrifugation, the culture supernatants (HAdV) and cell homogenates (BKPyV) were frozen in liquid nitrogen to preserve the native properties of the viruses. The presence of viruses were confirmed by means of a PCR reaction, with primers complementary to HAdVs and BKPyV DNA, as described in the section *‘*Plasma and urine screening examination for viral infections’. Before further experiments, a TCID-50 (the dose of virus that produces CPE in 50% of wells) was determined for each virus using monolayer cultures of A549 cells.

### A549 cell inoculation with HAdV5 and BKPyV

1 x 10^5^ of A549 cells per ml were cultured on 32 mm Petri dishes in a growth medium (Dulbecco’s Modified Eagle’s Medium supplemented with 5%, 1% penicillin-streptomycin) under stringent aseptic conditions, at 37 °C, 5% CO_2_. An MOI (multiplicity of infection; the average number of virus particles infecting each cell), was estimated by dividing the number of infectious particles by the number of cells inoculated with adenovirus or BKPyV. Since plaque forming units (PFU) (TCID- 50 x 0.7) represents the estimated number of infectious units per volume of virus material, the total number of virus particles used for inoculation was calculated. After 24 h, 90% confluent cells were inoculated for 2 h with HAdV5 (MOI 0.1), BKPyV (MOI 0.8) or both viruses (HAdV5 MOI; 0.1- removed after 2 h, and BKPyV MOI 0.8 for the following 1.5 h) in 2% maintenance medium (DMEM supplemented with 2% FBS and 1% of antibiotics). After inoculation, the medium containing virus/es was replaced with a new portion of fresh 2% maintaining medium. The culture were maintained for 2, 24 and 48 hours, respectively. The culture control was A549 cells cultured in the same conditions (the same culture plate) in growth and maintenance media without virus.

### Plasma and urine screening examination for viral infections

A screening examination of all clinical samples was performed with singleplex qualitative PCR assays, described previously [9]. Briefly, assays consist of HotStarTaq Master Mix Kit (QIAagen GmbH, Germany), forward and reverse primers (0.5 µM final conc.), double distilled water and 6 µl of target viral nucleic acid. Reference strains of human adenoviruses and BKPyV were used as positive controls in each run. Distilled water instead of template DNA served as a negative control. On the basis of a HAdV standard we concluded that it was possible to reliably detect 7.5 x 10^1^ copies of viral DNA per ml of the sample. Positive samples were quantified by quantitative real-time PCR (Rq-PCR) to determine virus load, as indicated below. Primers for HAdV and BKPyV assays are presented in Table 1.

**Table 1:** The oligonucleotide sequences

Target	Sequence 5′–3′	
	Forward	Reverse
HAdV	GCCGAGAAGGGCGTGCGCAGGTA	ATGACTTTTGAGGTGGATCCCATGGA
BKPyV	AGTCTTTAGGGGTCTTCTACC	GGTGCCAACCTATGGAACAG
HAdV5-*E4orf1*	TGCTAAAAAGCGACCGAAAT	GCGCTGTATGTTGTTCTGGA
BKPyV-*Tag*	TGC AAC TCT TGA CTA TGGGGG	AGG CTGGATTCTGAGATAAGTATG
BKPyV-*tAg*	ATTTTATCCTCGTCGCCCCC	GAGCTGCCTGGGGAAATCTT
*G6PD*	GCACAAGCTTCAGGTCTTCC	GAACAAGATCCGAGCGTAGC

### Measurement of HAdV load in clinical samples and culture collections

Virus copy number in clinical samples from patients and culture collections were quantified using a real-time PCR described previously [27]. Briefly, q-PCR was performed using the CFX 96^TM^ Real-Time System (BioRad, Hercules, USA). The reaction mixture consisted of 1 × TaqMan Universal Master Mix (Thermo Fisher Scientific, Waltham, USA), primers and probes as described in reference [27], 1.68 μl of 10 × Exo IPC Mix (exogenous internal positive control) (Thermo Fisher Scientific), 5 μl of target DNA and H_2_O-DEPC (diethylpyrocarbonate) to a final volume of 24 μl. Five μl of 50 × Exo IPC DNA (Thermo Fisher Scientific) was added into a lysis buffer prior to extraction of DNA and was co-amplified in the multiplex reaction as IPC in order to monitor the integrity of the DNA and the presence of PCR inhibitors. Appropriate no template controls (NTC), no amplification controls (NAC) and 3 different positive controls (PC, viral DNA) were used in each run.

### Analysis of gene expression

TRIZOL reagent (Thermo Fisher Scientific) was used to isolate total RNA from virus inoculated cells and from uninfected control cells, according to the manufacturer‘s instructions. 320 ng of pure RNA was reverse transcribed to cDNA with the iScript cDNA Synthesis Kit (BioRad). Relative q-PCR and CFX96 Touch (BioRad) were used for expression analysis of the following genes: HAdV5 *E4orf1* gene (early region four open reading frame one of HAdV), BKPyV *TAg* (large tumor antigen) and BKPyV *tAg* (small tumor antigen) compared to *G6PD* (glucose-6-phosphate dehydrogenase). Briefly, the reaction consisted of iTag Universal Sybr Green Supermix (BioRad), forward and reverse primers (250 nM final conc.), water and cDNA (320 ng) in a final volume of 20 µl. The sequences of primers are showed in Table 1. The amount of particular mRNAs relative to G6PD was calculated as 2^−ΔCt^. The relative expression of respective genes was compared in single or double virus infected cells.

### Statistical analysis

Statistical analysis was performed using GraphPad Prism (v. 5.0). Two-way ANOVA, Student’s t-tests or Mann-Whitney test were used, as appropriate. The correlation between HAdV5 and BKPyV genes was assessed by means of Spearman’s rank correlation test. P < 0.05 indicated statistical significance. Data are presented as the mean ± standard error (S.E.M).

## Results

### Level of HAdV replication in patients with co-infection

**Fig. 1:**
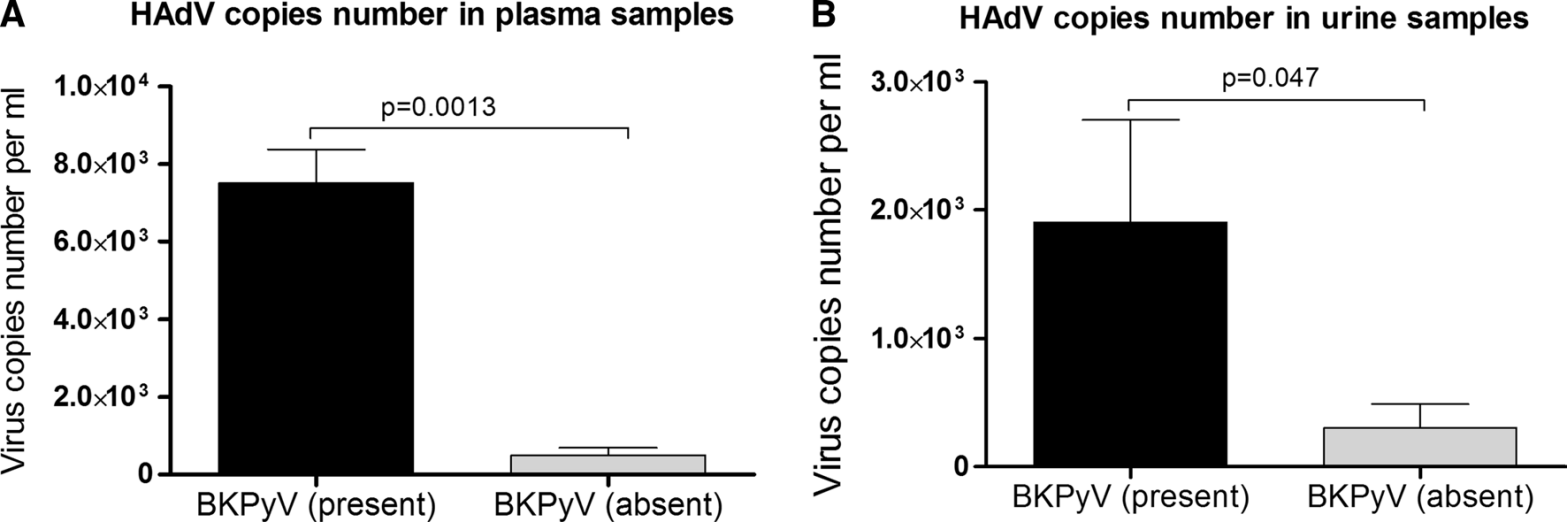
Human adenovirus 5 copy number in plasma (A) and urine (B) samples from patients co-infected or not with BKPyV. Mean ± S.E.M. Plasma n = 39, urine n = 113. BKPyV, polyomavirus BK; HAdV, human adenovirus

Multiple plasma and urine samples obtained from 156 patients undergoing hematopoietic stem cell transplantation were examined for the presence of concomitant infections of HAdV and BK polyomavirus. 71 patients (45.5%) were positive for HAdVs. 24 of them (33.8%) were also positive for BKPyV. Data showed that the copy number of HAdV was significantly higher in the plasma (mean 7.5 x 10^3^ ± 8.5 x 10^2^ copies/ml) and urine (mean 1.9 x 10^3^ ± 8.0 x 10^2^ copies/ml) of samples from patients with co-infection, than in the samples from patients with isolated HAdV infection: plasma (mean 4.9 x 10^2^ ± 1.9 x 10^2^ copies/ml) and urine (mean 3.2 x 10^2^ ± 2.0 x 10^2^ copies/ml) (Figure 1).

### Expression of *HAdV-E4orf1* gene during HAdV and BKPyV co-infection

**Fig. 2:**
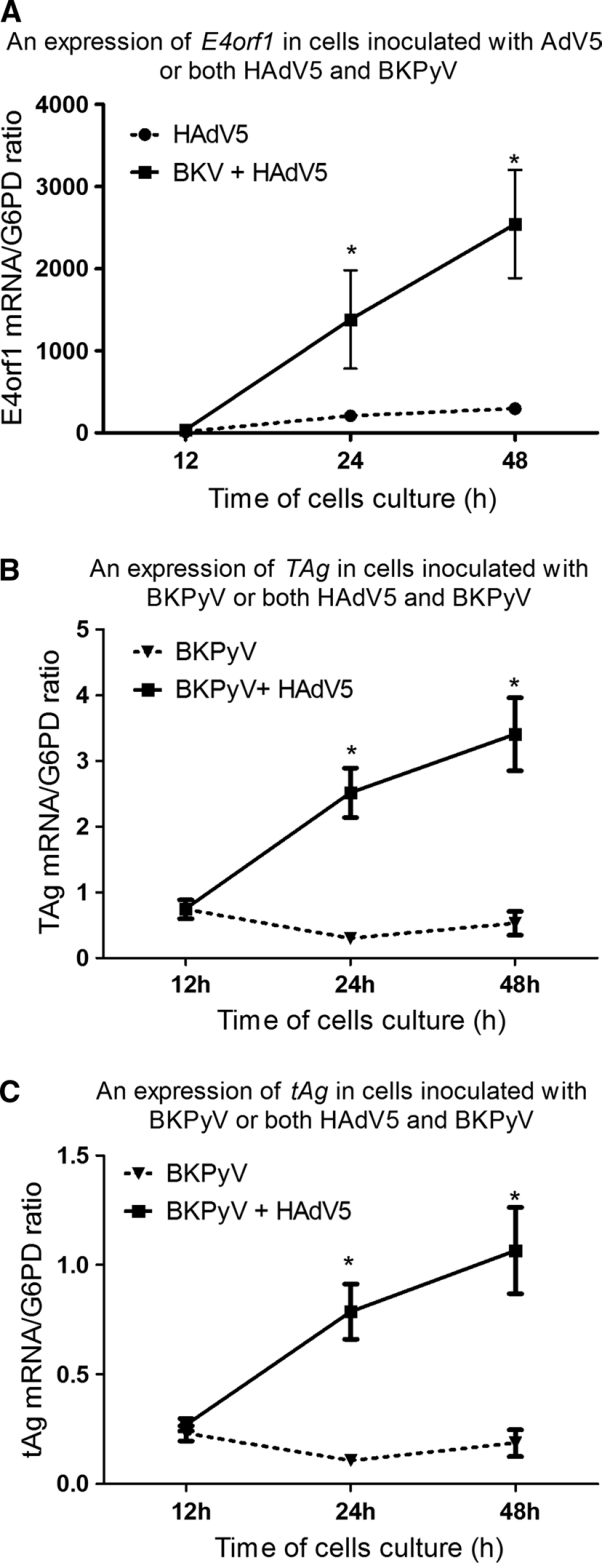
Expression of HAdV5 *E4orf1* (A) and BKPyV *TAg* (B) and *tAg* (C) genes in A549 cells inoculated with one or both viruses during 12-48 h of culturing. Mean ± S.E.M; N = 6/per group; A549, adenocarcinomic human alveolar basal epithelial cells; BKPyV, polyoma BK virus; *E4orf1*, open reading frame 1 of early region 4; *G6PD*, glucose-6-phosphate dehydrogenase; HAdV5, human adenovirus 5; *TAg*, large tumor antigen gene; *tAg*, small tumor antigen gene

To determine an interaction between HAdV and BKPyV in infected host cells, the expression of the HAdV5 replication gene (*E4orf1*) in the presence of BKPyV virus was assessed in cells inoculated with one or both viruses. Data showed that co-infection of adenocarcinomic human alveolar basal epithelial cells with human AdV5 and BKPyV led to an enhanced expression of the *E4orf1* gene during the time of cultivation. Expression of the *E4orf1* gene was increased 2.7 times after 12 hours, 6.7 times after 24 hours and 8.6 times after 48 hours of co-infection (Figure 2A), when compared to expression of *E4orf1* in cells inoculated with HAdV5 alone.

### Expression of BKPyV-*TAg* and BKPyV-*tAg* genes during HAdV and BKPyV co-infection

**Fig. 3:**
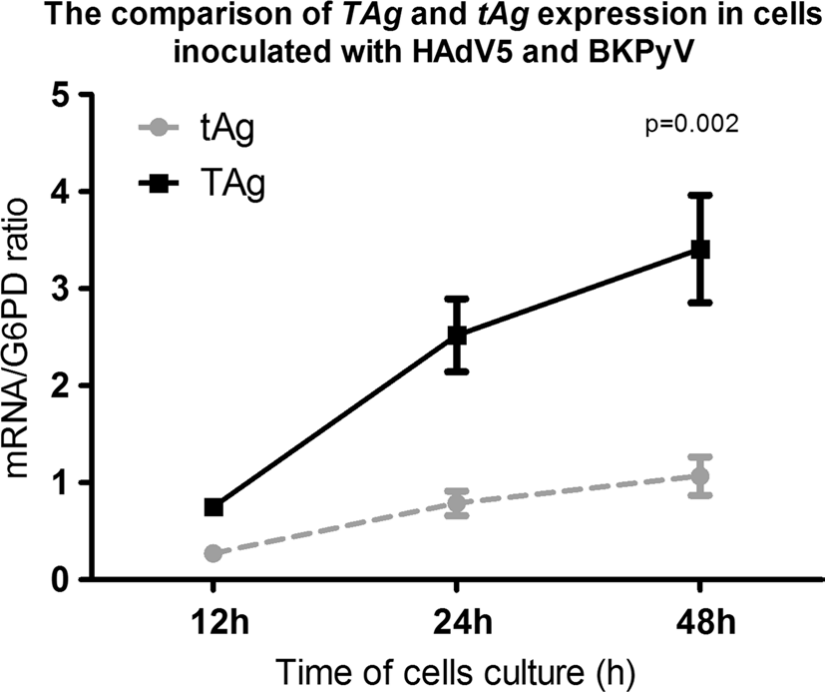
Comparison of BKPyV *TAg* and *tAg* gene expression in A549 cells inoculated with both viruses during 12-48 h of culturing. Mean ± S.E.M; N = 6/per group; A549, adenocarcinomic human alveolar basal epithelial cells; BKPyV, polyoma BK virus; *G6PD* glucose-6-phosphate dehydrogenase; HAdV5, human adenovirus 5; *TAg*, large tumor antigen gene; *tAg*, small tumor antigen gene

Similar to above, the expression of BKPyV genes such as *TAg* and *tAg* in the presence of HAdV5 was assessed and shown to be significantly higher in comparison to cells infected only with BKPy. Expression of *TAg* and *tAg* were increased approximately 8 and 6 times after 12 and 48 hours of co-infection, in comparison to their expression in the absence of adenovirus (Figure 3B-C). Data also showed that the expression of *tAg* was statistically lower during co-infection than expression of *TAg* (Figure 3).

### Correlation of *E4orf1* and *TAg/tAg* genes

**Fig. 4:**
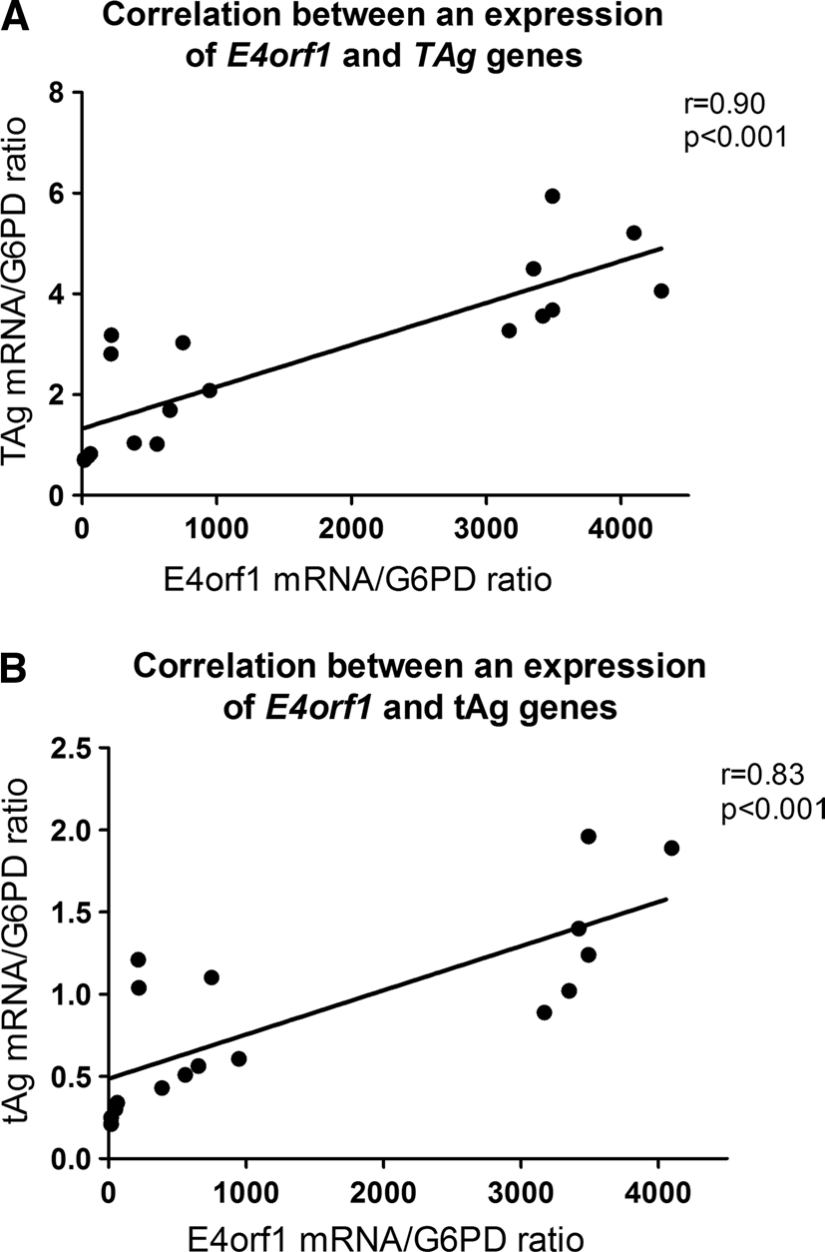
The correlation between HAdV5 *E4orf1* and BKPyV *TAg* (A) and *tAg* (B) gene expression in A549 cells inoculated with both viruses 48 h post-inoculation. N = 18/per group; A549, adenocarcinomic human alveolar basal epithelial cells; BKPyV, polyoma BK virus; *E4orf1*, open reading frame 1 of early region 4; *TAg*, large tumor antigen gene; *tAg*, small tumor antigen gene

Data showed that inoculation of A549 cells with adenovirus and BK polyomavirus led to the simultaneous replication of both viruses. Expression of adenovirus *E4orf1* gene positively correlated with expression of BKPyV-*TAg* (r = 0.90, p < 0.0001) and *tAg* (r = 0.83, p < 0.001) (Figure 4A-B).

### Adenovirus copy number in cells inoculated with BKPyV

**Fig. 5:**
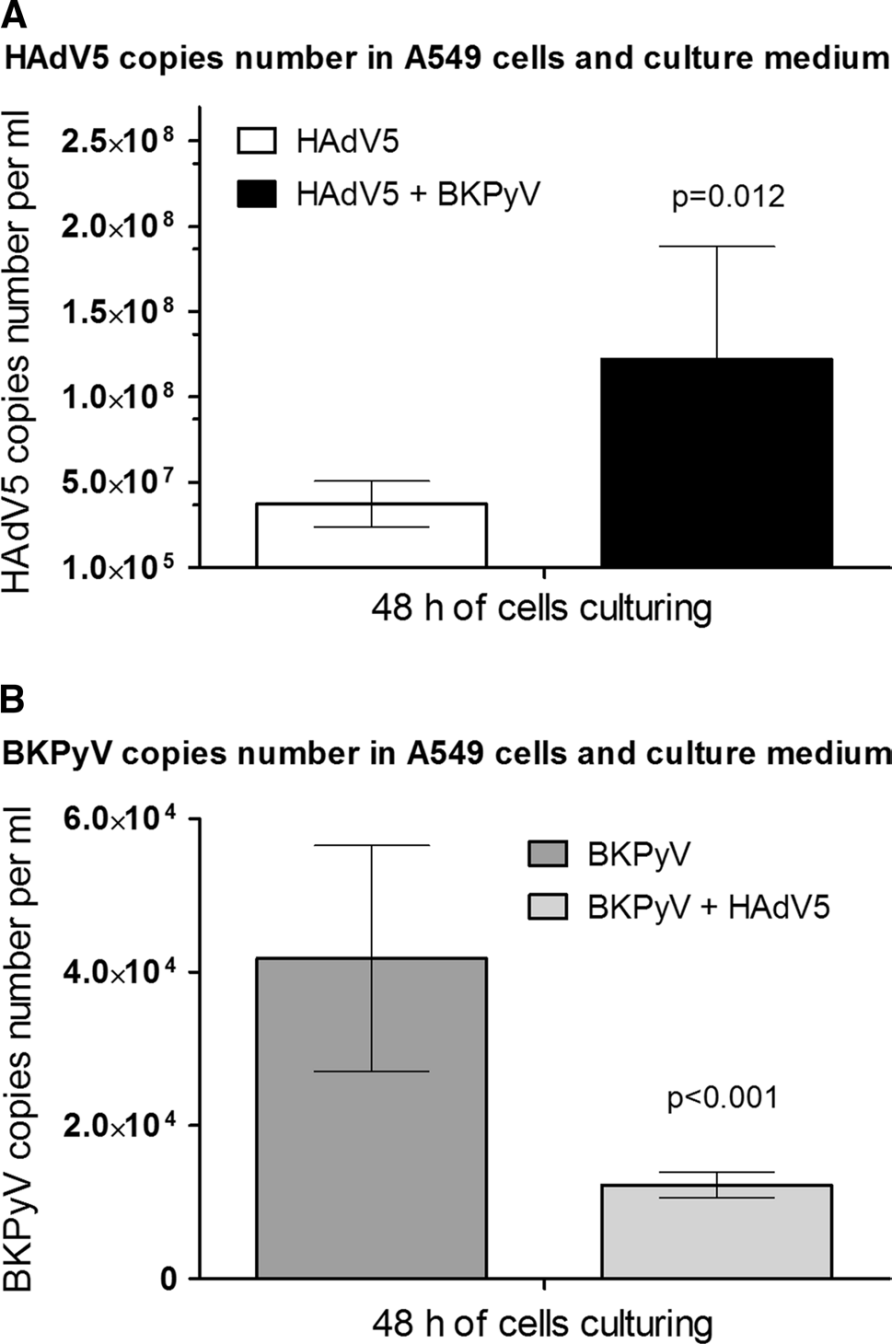
HAdV5 (A) and BKPyV (B) load in A549 homogenates and culture supernatants (taken together) 48 h post-inoculation in the presence, or absence, of co-infection. Mean ± S.E.M; N = 6; A549, adenocarcinomic human alveolar basal epithelial cells; BKPyV, polyoma BK virus; HAdV5, human adenovirus 5

To confirm that BK polyomavirus infection may potentiate the replication of HAdV, HAdV5 copy number in supernatants and A549 cells collected during culturing of HAdV5 and BKPyV were measured. HAdV5 copy number in HAdV5 and BKPyV co-inoculated cells and culture medium were significantly higher than in those cultures inoculated only with adenovirus (Fig. 5A). There was no enhanced replication of BKPyV seen in the presence of adenovirus (Fig. 5B)

## Discussion

The main aim of the current study was to examine whether co-infection of A549 cells with polyomavirus BK and human adenovirus 5 leads to an increased expression of adenovirus replication genes, which in turn facilitates the replication of adenovirus. We chose HAdV5 as a representative since they are prevalent in many geographic regions and in immunocompromised patients. We also showed that co-infection of HAdV5 and BKPyV in patients undergoing hematopoietic stem cell transplantation leads to elevated adenovirus viremia and viruria.

The phenomenon of co-infection occurs in a substantial number of patients with impaired immunity. A large group of patients with an unsuitable response to an infection are subjects who received immunosuppressive treatment due to underlying disease such as hematological malignances, solid organ transplantations, autoimmune and rheumatic diseases [28]. Relatively standardized immunosuppressive regimens following hematological stem cell transplantations in clinical practice define the predictable timetable of infections during the post-transplant course. Among them, HAdV and BKPyV infections are also a common complication during the first 6-12 months after transplantation [29, 30]. In the current study we observed that HAdV5 copy number in the plasma and urine samples of patients with co-infecting BKPyV was significantly higher than in samples without BK polyomavirus co-infection. This led to the hypothesis that the increased replication of HAdVs may be facilitated by co-infecting BKPyV. The phenomenon of transactivation of co-infecting viruses has been considered many years ago, but it is still not clear. It is known that interactions between co-infecting viruses might be due to trans-activation or repression of genes involved in regulatory processes of the host cell or indeed other viruses [23]. For example, it was shown that the adenovirus regulator protein E1A upregulates the cytomegalovirus major immediate early promoter (MIEP) [25]. In 1990 Tada et al. showed that the Tat protein of HIV-1 (human immunodeficiency virus 1) is able to trans-activate JC polyomavirus (JCV) late promoter which in turn led to the stimulation of JCV gene expression [31]. Two years later Moch et al. showed that human T-cell lymphotropic virus 1 strongly trans-activated MIEP [24]. There were also cases of BKPyV transactivation reported. Gendelman et al. (1986) showed that BKPyV trans-activates HIV long terminal repeats (LTR) in rhabdomyosarcoma cells [32] and Kristoffersen et al. (1997) published a paper in which transactivation of human cytomegalovirus by BKPyV T antigen was shown [26]. Taking into account the above research, our data showing an enhanced HAdV5 load in co-infected samples precipitated further analysis.

BK polyomavirus is a common, nonenveloped, double-stranded DNA virus that infects many species including humans [33]. In transplant recipients it can cause tubulointerstinal nephritis [34], ureteric stenosis [35], nephropathy [36] and hemorrhagic cystitis [37]. In recipients of HSCT BKPyV is able to cause a persistent infection (latency) in renal tubular epithelial cells and uroepithelium, leading to periodic reactivation and nephropathy. In some rare cases, viruria leads to viremia and disseminated disease [38]. The BKPyV genome consists of a circular double-stranded DNA genome containing nearly 5 Kb coding for at least seven viral proteins. The early proteins include the large tumor antigen (TAg), the small tumor antigen (tAg), and the recently discovered truncated tumor antigen (TruncTAg) [39]. TAg and tAg are the main early proteins, which are essential for both driving viral replication and inducing cellular transformation. They play a significant role in viral genome replication by regulating the entry of quiescent cells into the cell cycle and by autoregulation of viral early mRNA synthesis. They also participate in the modulation of cellular gene expression proceeding viral DNA replication [40, 41]. This virus’s potential for heterologous transcriptional transactivation has been established previously [42] but our study seems to be the first to investigate the influence of BKPyV co-infection on gene expression and replication of adenoviruses in immunocompromised subjects. We showed that the *TAg* and *tAg* genes of BKPyV and the *E4orf1* gene of HAdV5 are highly expressed in A549 cells inoculated with both viruses in comparison to cells inoculated with only one virus. An increasing expression ratio over time means that co-these infecting viruses likely express early genes involved in the early phase of virus replication in permissive cells. This observation is consistent with the findings of Heilbronn et al. (1993) who demonstrated that JC polyomavirus was able to replicate in potentially non-permissive fibroblasts when co-infected with HCMV [43]. However, Kristoffersen et al. (1997) showed no increased expression of BKPyV *TAg* mRNA or protein in A459 cells co-infected with HCMV. Here we demonstrate that co-infection of both adenovirus and BK polyomavirus led to enhanced expression of immediate early genes of both viruses. Enhanced mRNA levels in cells inoculated with both viruses, in comparison to cells inoculated with one virus, proves that co-existence of HAdV5 and BKPyV facilitates the early phase of these viruses replication [26].

In further analysis we showed that expression of the HAdV5-*E4orf1* gene strongly correlated with the expression of *TAg* and *tAg*. These data showed that the increased expression of BKPyV early genes was accompanied by increased expression of an adenovirus transcription unit which is transcribed early in the viral reproductive cycle. E4orf1 is mostly involved in the regulation of viral transcription, in replication of viral DNA, and in suppression of the host response to infections [44]. We also showed that the expression of TAg, which is able to trans-activate heterologous virus promoters [42] was expressed much more intensively than tAg, which is mainly associated with lytic cycle. This may suggest that the expression of *TAg* is a leading factor which affects adenovirus replication.

Enhanced replication of HAdV5 in the presence of BKPyV may lead to increased virulence in adenovirus infections and serious consequences in immunocompromised patients [3, 45]. Enhanced expression of *E4orf1* in cells inoculated with HAdV5 and BKPyV was associated with an increased replication of HAdV5. Significantly higher HAdV5, but not BKPyV, load in cells and culture media in the presence of both viruses provides evidence that enhanced expression of *TAg* and *tAg* is associated with increased expression of *E4orf1* and facilitates adenovirus replication. However, our data did not show that adenoviruses are able to enhance BKPyV replication.

**Fig. 6:**
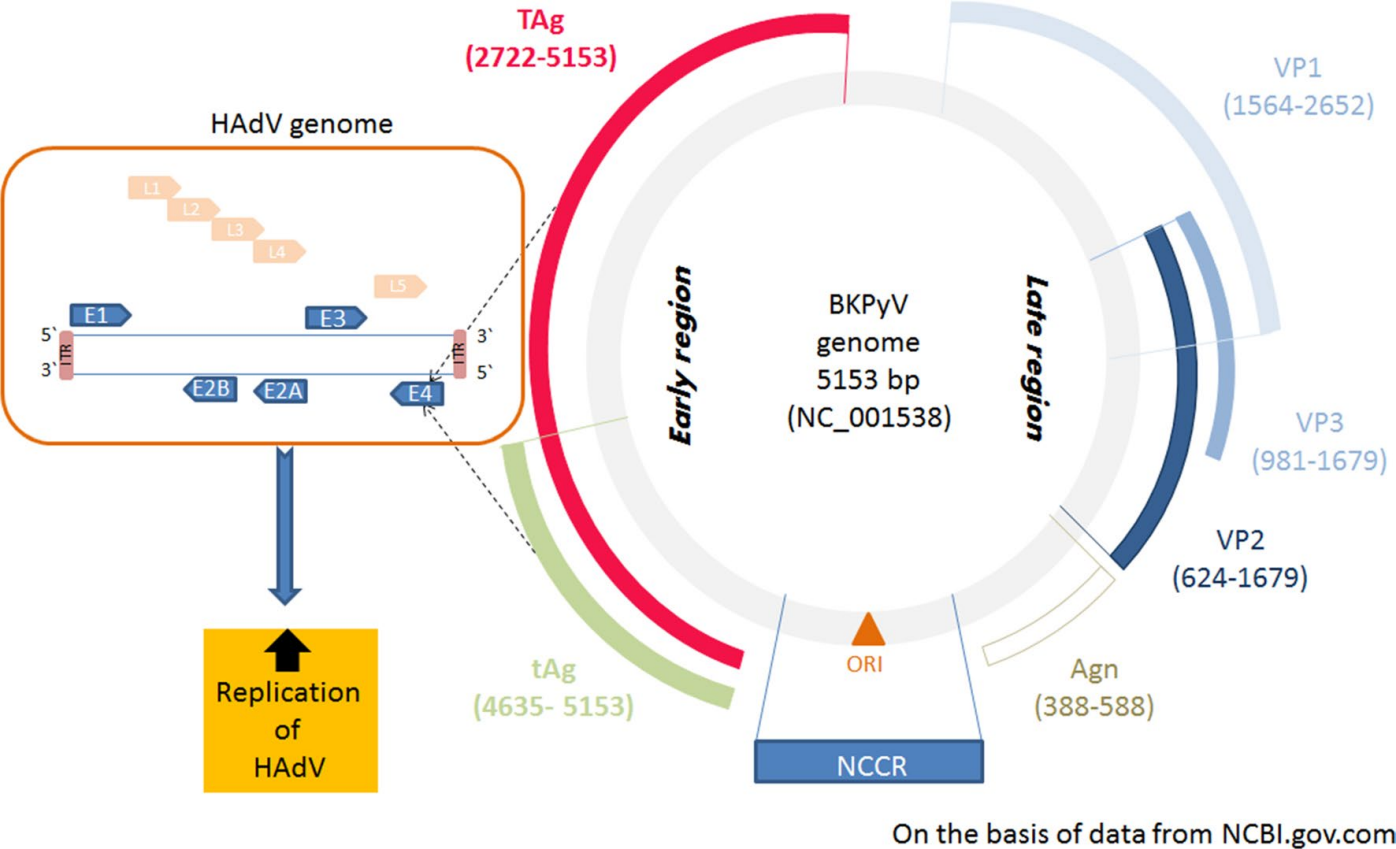
An increased expression of *TAg* and *tAg* is accompanied by increased expression of *E4orf1*, leading to enhanced replication of human adenovirus. Agn, agnoprotein; AgT, large T antigen; Agt, small T antigen; E1-*E4*, genes for early proteins 1-4; *E4orf1*, open reading frame 1 of early region 4; ITR, inverted terminal repeats; *L1-L5*, genes for late proteins; NCCR, noncoding control region; ORI, origin of replication; *VP1-3*, genes for late capsid proteins 1-3

In conclusion, since both adenoviruses and polyomaviruses are able to enter long term latency following primary infection and both are very common in patients undergoing hematopoietic stem cell transplantation [4, 20, 46–48], enhanced replication of HAdV due to BKVPyV co-infection seems to be alarming and may have an adverse effect for immunocompromised patients. We are aware that increased replication of adenovirus in patients with co-infection is, at least in a part, a result of deep immune deficiency. However, co-infection of adenovirus and polyomavirus BK in permissive cells proved that the presence of BKPyV increased replication of HAdV5 and viral load. For this reason co-infections in immunocompromised patients should be always considered as undesirable and likely to worsen the clinical course and outcome. Finally, we would like to highlight that the data obtained from this study was the basis for a new concept of HAdV5 and BKPyV trans-activation (Figure 6), which will be the main aim of future studies in our lab based on plasmid transfection.
